# A study on health status of women engaged in a home-based “Papad-making” industry in a slum area of Kolkata

**DOI:** 10.4103/0019-5278.40814

**Published:** 2008-04

**Authors:** Sima Roy, Aparajita Dasgupta

**Affiliations:** Department of Community Medicine, Institute of Postgraduate Medical Education and Research, All India Institute of Hygiene and Public Health, Kolkata, India; 1 Department of Preventive and Social Medicine, All India Institute of Hygiene and Public Health, Kolkata, India

**Keywords:** Felt needs, health, ‘papad’ industry, women

## Abstract

**Background:**

The ‘papad-making’ industries of India have provided ample opportunity of employment for the women workers of low socio-economic class although their problems are not much explored. In this study an attempt had been made for the same.

**Aims:**

1. To find out the health status of the women. 2. To find out the factors, in the working conditions, influencing their health status. 3. To assess their felt needs.

**Settings:**

A slum area of Kolkata.

**Design:**

A cross-sectional, descriptive type of observational study.

**Methods and Materials:**

The slum was chosen by random sampling method. Following this, complete enumeration method was adopted. Data were collected by interview and clinical examination of the women engaged in this occupation with a predesigned and pretested schedule.

**Statistical Analysis:**

Proportions and *Chi-square* test.

**Results:**

77.5% were in the reproductive age group and none were below 14 years. Most of them belonged to poor socioeconomic status. Sixty per cent were in this occupation for more than 10 years and they spent 5 hours for this work daily over and above their household job. Musculoskeletal problem was their commonest health problem. Pallor, angular stomatitis, pedal edema, chronic energy deficiency were found on examination. Personal hygienic measures taken were far from satisfactory. A focus group discussion revealed their health and family problems, dissatisfaction about their working conditions and wage. Other needs identified were home visits for their health care, free medicines and health education.

**Conclusion:**

Need exists for a participatory occupational health programme for this working population.

## INTRODUCTION

In developing countries, great efforts are directed towards the development of small-scale industries as the engine for their economic growth. According to WHO over 1000 million people worldwide are employed in small-scale industries.[1] The “papad-making” industry of India is one such industry which has provided ample opportunity of employment for the women of low socio-economic class.

Shri Mahila Griha Udyog Lijjat Papad is a ‘papad-making’ industry of India which manufactures ‘papad’ along with various other products.[2] It is primarily a cottage industry.[3]

The ‘papad’ is a thin South Asian wafer, sometimes described as a cracker or flatbread. It is made from pulses or rice flour. Salt and peanut oil are added to the flour to make a dough, which can be flavored with chili, cumin, garlic or black pepper. Sometimes baking soda is also added. The dough is rolled manually (10) into a thin, round flat bread and then dried (traditionally in the sun) and can be cooked by deep-frying, roasting over an open flame, toasting or microwaving, depending on the desired texture. ‘Papad’ can be of different variety depending upon the type of pulses or flavors used like moong (50% udad and 50% moong), moong special (50% udad and 50% moong), punjabi masala special, udad special, udad with garlic and chillies, udad with pepper, udad with red chillies.[4]

Although such industries are identified with women empowerment in India,[5] the employees are found to have not subjected to occupational health-and-safety provisions. As a result they suffer adverse health impacts.[1]

In this study an attempt has been made to find out the health status of the women engaged in a ‘papad-making’ industry, the occupational factors influencing their health status and their felt health needs.

## MATERIALS AND METHODS

It was a cross-sectional study carried out in a slum area of Kolkata where there is a concentration of women engaged in the ‘papad-making’ industry. The slum area is inhabited by people of poor socioeconomic status. The study population consisted of women engaged in ‘papad-making’ industry. The particular slum where the study was carried out was chosen by random sampling method. Following this, complete enumeration method was adopted. Door to door survey was done and whenever a women engaged in ‘papad-making’ industry was found relevant, information was collected by interviewing the lady, by clinical examination and by observation. The sample size was 80. It consisted of women between the age of 14-60 yrs. The study tool consisted of predesigned and pretested schedule with questions regarding their socio-economic condition, their occupational history and their health problems.

## RESULTS

Table 1 shows that maximum number of the study population (77.5%) are in the reproductive age group and none are below 14 years. 82.5% are married, 87.5% are literate, 77.5% belong to the nuclear family and 78.8% of the married women had two or less than two children. Most of them belong to poor socioeconomic status.

**Table 1:** Socio-demographic profile of the study population (*n* = 80)

	No.	%
Age range (years)		
15-45	62	77.5
45-60	18	22.5
Marital status		
Married	66	82.5
Unmarried	14	17.5
Educational status		
Literate	70	87.5
Illiterate	10	12.5
Type of family		
Nuclear	62	77.5
Joint	18	22.5
No. of children (among married women)		
<2	52	78.8
>2	14	21.2
Family income		
3000-4000	22	27.5
2000-2999	44	55
1000-1999	10	12.5
800-999	4	5

From Table 2 it is evident that 60% of the women are in this occupation for more than ten years and most of them spend about five hours for the ‘papad’ making work over and above their household job.

**Table 2:** Occupation related information (*n* = 80)

	No.	%
Duration of work (in years)		
<5	16	20
5-10	16	20
>10	48	60
Hours of work per day		
<5	18	22.5
>5	62	77.5

Table 3 shows the health profile of the study population. Musculoskeletal problem is their commonest health problem. Neck is the most commonly affected part followed by the low back [Table 4]. A statistically significant relationship (χ^2^ = 20.11, df = 1, *P* = < .001) was found to exist between duration of occupation and musculoskeletal problem [Table 5]. Their other problems include generalized weakness, acidity, menstrual problems, insomnia, headache, excessive sweating, burning sensation during micturition, swelling of feet and problem with vision. Pallor (75%), angular stomatitis (25%), pedal edema (17.5%), poor oral health (15%), hypertension (12.5%), epigastric tenderness (10%), scabies (7.5%) were found on examination [Table 3].

**Table 3:** Health problems of the study population (*n* = 80) multiple response

	No.	%
Health problems as stated		
Musculoskeletal problems	66	82.5
Generalized weakness	20	25
Acidity and heart burn	10	12.5
Menstrual problems	10	12.5
Insomnia	2	2.5
Headache	2	2.5
Excessive sweating	2	2.5
Burning sensation during micturition	2	2.5
Swelling of feet	2	2.5
Problem with vision	2	2.5
Findings on examination		
Pallor	60	75
Angular stomatitis	20	25
Edema	14	17.5
Poor oral health	12	15
Hypertension	10	12.5
Tenderness in epigastric region	8	10
Scabies	6	7.5

**Table 4:** Body parts affected with musculoskeletal problems (*n* = 66) multiple response

Parts affected with musculoskeletal problems	No.	%
Neck	22	33.33
Low back	20	30.30
Knee	12	18.18
Shoulder	12	18.18
Elbow	10	15.15
Forearm	10	15.15
Leg	6	9.09
Ankle	6	9.09
Upper arm	4	6.06

**Table 5:** Relationship between duration of occupation and musculoskeletal problems (*n* = 80)

Duration of occupation	Musculoskeletal symptoms present	Musculoskeletal symptoms absent	Total
<5 yrs	6	10	16
>5 yrs	57	7	64
Total	63	17	80

χ^2^ = 20.11, df = 1, *P* = < 0.001, significant

Table 6 gives us an idea of the status of personal hygiene of the study population. The ablution habit of the study population is not satisfactory. 15% of the study population did not use soap to wash hands after defecation. On the contrary it was observed that the women preparing the papad do not wash their hands before preparing the papad (71.3%). Moreover they do not pair their nails properly (78.7%). It was observed that the papad are dried in the open passages of the slum area where there are open drains teeming with flies and mosquitoes and the storage practice is also unhygienic.

**Table 6:** Status of personal hygiene of the study population (*n* = 80)

	No.	%
Ablution habit		
Use soap	68	85
Do not use soap	12	15
Washing hands		
Wash	23	28.7
Do not washes	57	71.3
Pairing of nails		
Nails paired	17	21.3
Nails not paired	63	78.7
Drying and storage of Papad		
Under insanitory conditions	80	100
Under sanitary condition	None	

## DISCUSSION

Other than the home environment, the workplace is the setting in which many people spend the largest proportion of their time. But for many people, particularly in developing countries, the boundary between their home and workplace environments is blurred, since they often undertake agricultural or cottage industry activities within the home.

In favorable circumstances, work contributes to good health and economic achievements. However, the work environment exposes many workers to health hazards that contribute to injuries, respiratory diseases, cancer, musculoskeletal disorders, reproductive disorders, cardiovascular diseases, mental and neurological illnesses, eye damage and hearing loss, as well as to communicable diseases.

The informal sector and small-scale industries, in particular, are subject to numerous workplace hazards[1] and health hazards of women workers require special mention and have always been traditionally under-estimated.[6]

The women engaged in this ‘papad-making’ industry bring the kneaded flour home and prepare the ‘papad’ at home and deliver it to the employer. No machinery is used at the production level and everything is done manually.[7] Since they belong to low socio-economic class and prepare the ‘papad’ at home, they have to carry out all their household jobs along with this specific job. As a result their duration of work is more than the housewives as well as the women workers working outside.

In this descriptive type of observational study an attempt has been made to find out the health problems of the ‘papad-making’ women workers, the occupational factors influencing their health status and their felt health needs.

Musculoskeletal problem is the commonest health problem of the study population. Canadian women's health network has reported that musculoskeletal disorders are the most serious hazards of working women.[8]

Neck is found to be the most commonly affected part followed by the low back. Study done by How-Ran Guo reported that musculoskeletal disorders of body parts other than the back are the neck, shoulders, hands and wrists.[9]

In the ‘papad-making’ industry there is no provision for a retirement age, as the emphasis is on earning one's bread through daily work, all through one's life.[10] Although such principle of this industry go well with the self-sufficiency of women, it however increases the duration of occupation and increases the possibility of suffering more from musculoskeletal problem. A statistically significant relationship (χ^2^ = 20.11, df = 1, *P* = < .001) was found to exist between duration of occupation and musculoskeletal problem. Similar findings can be observed in other studies. A study by Brhel *et al*., revealed that repetitive strains of upper extremity for 19.9 + 9.3 yrs resulted in carpal tunnel syndrome in a group of workers.[11] Forsmann *et al*., reported that overuse of upper extremity results in shoulder myalgia.[12] Frost *et al*., reported that shoulder intensive work is a risk factor for impingement syndrome of the shoulder.[13] That the risk of developing musculoskeletal disorders from an activity depends on the frequency, duration and physical demands of the activity is also reported by ILO.[6] Various population based surveys have also shown positive associations between musculoskeletal disorders and work factors like awkward postures, high physical exertion and vibration.[14]

Papad being a food item needs to be handled hygienically. In Lijjat industry care is taken for quality assurance[3] moreover surprise visits are made to various branches to assure that production conditions are hygienic.[7] Orientation courses in cooking and hygiene are given along with many other vocational training.[15] But unfortunately the personal hygienic status of the study population was far from satisfactory.

A focus group discussion was arranged for two days with 10 participants on the first day and 20 participants on the second day and their social, familial and personal problems were elicited. They complained of low wage, and lack of cooperation of the employer. Since they have to perform their household activities over and above this specific job, family care was affected, there was lack of time to take rest, to attend to personal health problems, to attend social programme and there is also no time for relaxation. During monsoon they have to use their own fuel to dry the “papad”, a problem which was also identified in Lijjat industry.[3] All of them consider that they are poorly paid and their pay must increase. Other needs identified are home visits for their health care and free medicines for their health problems.

Stress at work is a growing problem for all workers, including women. Many job conditions contribute to stress among women. Such job conditions include heavy workload, job insecurity, poor relationship with the supervisors, work that is repetitive and monotonous. Other factors such as work and family balance issues may also be stressors for women in the workplace.[16] Moreover the women are more likely to have difficulty in taking breaks, days off or holidays as reported by the European Foundation's 1996 European Union-wide survey.[17]

In recent times, contribution of poor work environmental conditions, poor perception of work conditions and presence of adverse health condition in workers on occupational injury occurrence has been highlighted. Nature of workplaces being varied determinants of occupational injury causation has also been different and identification of such responsible factors in relation to a specific work environment would not only help in exploring the etiology but also would be useful in planning prevention.[18]
